# Lesioning Through a Directional Deep Brain Stimulation Lead in the Subthalamic Nucleus

**DOI:** 10.5334/tohm.993

**Published:** 2025-04-07

**Authors:** Alfonso Enrique Martinez Nunez, Dorian M. Kusyk, Joshua K. Wong, Michael S. Okun, Justin D. Hilliard

**Affiliations:** 1 Norman Fixel Institute for Neurological Disorders, Departments of Neurology and Neurosurgery, University of Florida, Gainesville, FL, US

**Keywords:** Deep Brain Stimulation, Subthalamotomy, Hardware complication, Subthalamic nucleus

## Abstract

**Clinical Vignette:**

A 59-year-old woman with a previous subthalamic nucleus deep brain stimulation (DBS) implanted for Parkinson’s disease developed a hardware related infection.

**Clinical Dilemma:**

Wound dehiscence and infection developed and necessitated removal of the DBS system. The patient experienced excellent therapeutic benefit from her DBS and expressed concern about device removal.

**Clinical Solution:**

The patient was offered the option of a lesioning procedure which could be performed during hardware explantation. An operative procedure was conducted where the intracranial DBS lead was connected to a radiofrequency system in a deliberate effort to create a targeted subthalamotomy through the existing DBS lead. A multilevel lesion was generated using the contacts on the directional DBS lead. Following the lesion the DBS lead and hardware were removed.

**Gap in Knowledge:**

Creating a lesion through a DBS lead using radiofrequency ablation is a therapeutic option for patients not interested in later re-implantation or for those with a history of multiple DBS related infections. Lesioning through segmented leads introduces more complexity into the procedure.

## Clinical Vignette

A 59-year-old woman with Parkinson’s disease (PD) had disabling right hand tremor. The tremor was her first symptom and occurred six years prior to her current presentation. Her symptoms progressed and her tremor was demonstrated to be levodopa resistant. She also developed motor fluctuations, worsening bilateral symptoms as well as foot dystonia. Following aggressive attempts at medication optimization, she was offered DBS of the bilateral subthalamic nuclei (STN).

She was implanted with a Medtronic Sensight B33005 directional lead (Medtronic, Minneapolis, MN) into the left STN. This DBS lead model has four contacts. The second and third contacts on the DBS lead (contact 1 and 2) are segmented into three parts each, facilitating directional stimulation. Six months later, another lead was implanted in the right STN. Both were connected to a single Medtronic Percept PC pulse generator (Medtronic, Minneapolis, MN). She reported excellent tremor and bradykinesia improvements in the right hemibody with a monopolar omni-directional DBS setting (contact 1 negative, amplitude 1.5 mA pulse width 90 microseconds, and a frequency of 125 Hz). Clinic based programming through contact 2 revealed a similar therapeutic effect as revealed when activating contact 1. Based on a post-operative monopolar review (Table 1) and on imaging, her lead was judged to be in a reasonable location and the clinical benefit was excellent.

**Table 1:** Post-operative monopolar review. The thresholds for sensory and motor side effects are appropriate and suggest an adequate position within the STN.


	LEFT LEAD	RIGHT LEAD

Contact 0	0.9 mA (hand dysesthesia, anxiety)	1.4 mA (dysarthria)

Contact 1	1.4 mA (double vision and hand dysesthesia)	1.4 mA (face and hand contraction)

Contact 2	2 mA (hemibody tingling)	1.4 mA (face and hand contraction)

Contact 3	3 mA (hand and face contraction)	1.5 mA (face and hand contraction)


Six months following her second lead implantation, she fell and hit her head on a cabinet. Approximately one week following the fall, her left frontal incision was opened, and it manifested in a full thickness wound dehiscence. The DBS hardware on that side was exposed and the wound was observed to be draining.

## Clinical Dilemma

The DBS lead, the connector, and the neurostimulator all required explantation. The literature collectively has shown that attempts to salvage hardware have been associated with a low success rate when associated with an open wound [4].

The options we offered to the patient included: 1) Explanting the lead, culturing the organism and treating with a course of narrow-spectrum antibiotics; this option would require replacing the DBS lead with a follow-up surgical procedure. 2) Explanting the lead and replacing with another DBS lead in a different target, such as the globus pallidus, and following this approach with antibiotics. 3) Using the existing DBS lead to create a lesion prior to explantation. 4) Explanting the infected lead and offering a follow-up classical or focused ultrasound subthalamotomy.

## Clinical Solution

Explanting the DBS lead and treating with antibiotics is the most common approach currently used in clinical practice. Re-implantation of the DBS lead in the same target would be desirable only if there were satisfactory benefits. Switching to another brain target would be desirable if it was felt there was a suboptimal benefit or another symptom may be better addressed with a different target. In this case, the patient had a discussion with her neurosurgeon about the four possible approaches for management. She opted for a single lesioning procedure through the STN DBS lead. We chose this option because stimulation in this area has demonstrated symptom relief; therefore, creating a lesion at the same site could achieve similar results, eliminating the need for additional surgery.

**Figure 1:**
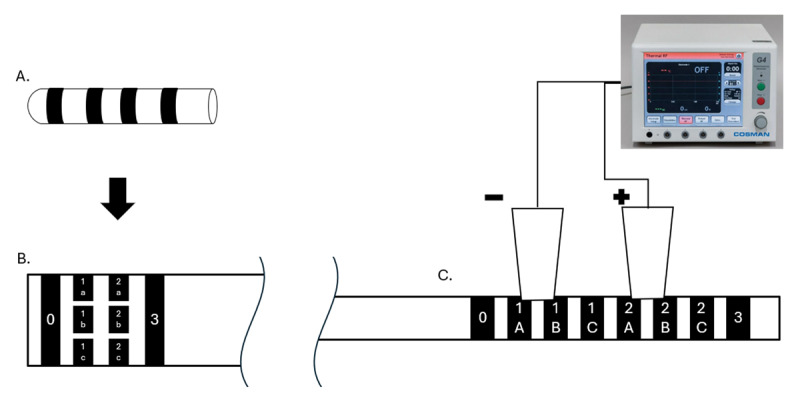
Directional DBS lead diagram **(A)**. When the lead is “unraveled” it has a total of 8 contacts – two monopolar levels and 2 levels with 3 segments each **(B)**. At the proximal end of the lead, where it would normally connect to the extension cable **(C.)** the 8 contacts are now split into 8 sequential contacts. This is where the alligator clips (represented by white trapezoids) of the radiofrequency ablation device connect for the lesioning procedure **(D)**. Note that each alligator clip covered two contacts due to its width (e.g. 1A and 1B, or 2B and 2C).

Creating a lesion through the DBS lead requires expertise, equipment, and real-time examination. The patient was kept awake, which facilitated continuous neurological assessment (Video 1). The contact or contacts providing the most benefit are typically selected for lesioning. In this case it was contacts 1 and 2, which corresponded to six total contact segments (3 segments on each contact). Multiple lesions were generated using permutations of these contacts as cathodes and anodes. We used the Cosman radiofrequency system (Optimus Medical Limited, Great Britain) and attached it to the proximal contact connectors via alligator clips, and by using a wiring diagram which corresponded to the location of the contacts, where each one of these proximal contact connectors corresponds to a segment or a contact in the stimulating end of the lead (Figure 1). The width of each alligator style clip covered two DBS contacts, so testing and lesioning were performed using two adjacent pairs of contacts (e.g. 1A and 1B both acting as either the cathode or the anode). Although the Cosman system can provide a monopolar charge, we used a bipolar configuration opting for tighter spatial control of the resulting lesion given the small size of the brain target.

**Video 1:** Intra-operative recording while creating a lesion. We show impedance monitoring, neurological testing, and finalization of the lesion.

**Figure 2:**
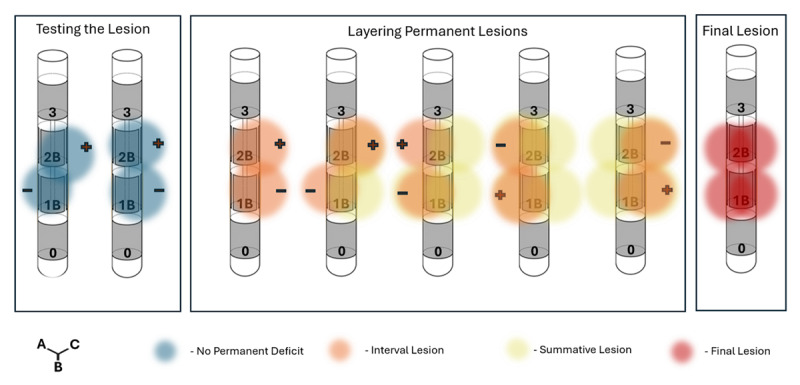
A graphical representation of the radiofrequency ablation procedure is demonstrated through a segmented lead. Left panel: a test lesion was made stimulating from contact segments 1A and 1B, followed by 1B and 1C. Middle panel: since there were no neurological deficits after the test lesion, we began to shape the permanent lesion. We set the stimulation at a higher amplitude in different combinations of segment pairs to cover the tissue surrounding contacts 1 and 2. Right panel: The final lesion spans the area surrounding all segments comprising contacts 1 and 2.

The Medtronic Sensight B33005 lead is composed of four contacts, of which the middle two contacts can be divided into three segments. We used this fixed property of the DBS lead to ensure the lesion was shaped in an omnidirectional pattern (Figure 2). Any commercially available DBS lead can be used as a vehicle to place a lesion in the brain. A test lesion was performed delivering 25 mA through contacts 1A and 1B for 60 seconds while continuously examining the patient for the possibility of a facial droop, dysarthria, and hemiparesis (Video 1). This process was repeated using contacts 1B and 1C as the cathodes and the 2B and 2C contacts as the reference electrodes (see Figure 2). Following the test lesion, 60 mA of current was applied for 60 seconds in each of the previously tested configurations. During the generation of the lesions, the impedance was gradually diminished, as the tissue surrounding the electrode was coagulated. Then we monitored for a rapid reversal in hardware impedance until we observed an open circuit as measured by the Cosman system readout. The lesion was extended using contact 2 as the cathodes and contacts on level 1 as the reference (see Figure 2, middle panel).

We then observed for tremor suppression, as well as performed a real-time examination for dysarthria, facial weakness, and possible hemiparesis during test and actual lesioning. There were no residual motor deficits, and there was coincident improvement of tremor, rigidity, and bradykinesia.

**Figure 3:**
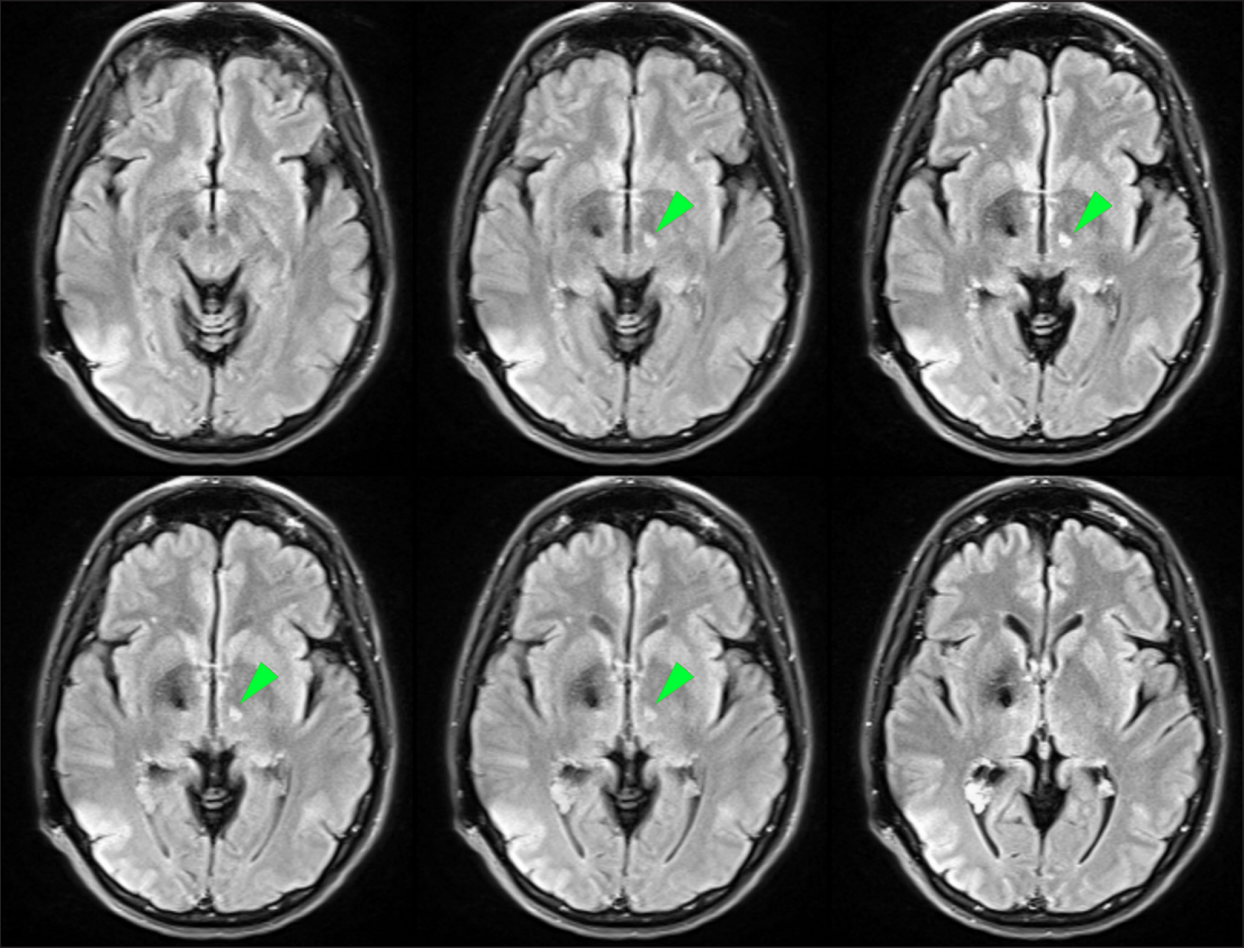
Axial sections of a brain MRI FLAIR sequence showing the lesion one-month post-procedure. The lesion can be seen on the left hemisphere as a hyperintense signal in the subthalamic region (green arrow heads).

The incision was temporarily closed, and the patient sedated for the second portion of the case, which involved removal of the intracranial DBS lead and repair of the burr hole cover. A small piece of coagulated brain tissue was observed at contacts 1C and 2C following lead removal, and this was consistent with a left subthalamotomy lesion (Figure 3). The left frontal wound was debrided, and revised. Intraoperative cultures grew *Enterobacter spp* and she was treated with home intravenous cefepime infusions.

Four weeks after the subthalamotomy, she had improvement of her right-sided tremor, rigidity, and bradykinesia. The improvements closely mirrored her previous DBS benefit. Her right-hemibody only Unified Parkinson’s disease Rating Scale part 3 score was 12 points prior to DBS surgery, improved to 6 after the left STN lead, and was also scored as 6 points improved following the subthalamotomy. There was no residual dysarthria or hemifacial weakness following the subthalamotomy that was detected by the clinical team or the patient.

Around 8 weeks after the surgery she reported new onset right side choreiform movements. Her daily levodopa dose was decreased and this led to resolution of the hyperkinesia. Most cases of optimally placed subthalamotomy lesions have been reported to be associated with long term outcomes up to six years of follow up [1].

## Gap in Knowledge

The proportion of DBS leads revised or explanted over a lifetime has been estimated to be 15–34% [3]. Common reasons for DBS explantation include infection, suboptimally placed leads with lack of therapeutic benefit, and hardware malfunctions including lead fracture [2,3]. In our case, the clinical benefit was excellent. Following a careful discussion with the neurosurgeon, our patient preferred to seek the possibility of benefit without the need for a future surgical procedure. There was no need for a new burr hole, another brain penetration with a DBS lead, or for trajectory planning. In this case, the DBS lead was optimally positioned and there were generous thresholds for benefit and side effects (Table 1). One important point if considering this approach is if a DBS lead is reasonably well placed and far enough away from internal capsule to avoid a persistent side effect.

Our patient required only unilateral intervention, and thus she kept her contralateral DBS lead in place. In the case of a person with a bilateral complication, such as in twiddler syndrome or some hardware infections, a second lesion is possible, however may be associated with more adverse events. Bilateral lesions have been associated with a higher risk of dysarthria, dysphagia, cognitive and gait disorders [1].

## Expert commentary

DBS leads are commonly removed for a multitude of reasons. It is important for clinicians to discuss the risks and benefits of all available options, inclusive of lesioning through an existing DBS lead. When the DBS lead is directional, the lesioning procedure is more complex, since multiple combinations of segments on the DBS lead can be utilized in order to achieve a robust omnidirectional lesion.

Lesioning through an existing DBS lead should always be performed using appropriate caution. Real-time examination during the procedure is critical to minimize the risk of adverse effects. The examination offers an opportunity to discontinue the lesion, thus decreasing the chances for a permanent deficit. Surgeons should be aware that when using an existing DBS lead, the temperature cannot be monitored as is employed during a traditional lesion probe. The surgeon has less control over the size of the lesion made through an existing DBS lead. This concern can be mitigated in cases when the DBS lead has been optimally placed and the thresholds for benefit and side effect shown to be robust. When DBS contacts contain segments, it can be tricky to make large lesions. It is thus possible that if the lesions are too small, they will prove suboptimal in the long term. Surgeons should, when possible, consider extending the lesion vertically, or lesioning through 2 different contacts on the DBS lead, as was performed in this case. Finally, though an omnidirectional lesioning approach was pursued in this case, it is possible to sculpt smaller lesions. These smaller lesions could be devised to avoid contacts too close to the internal capsule (or other structures) in cases where the DBS leads are suboptimally placed. It is, however, unclear if these smaller lesions will provide the same robust short- and long-term benefits.

## Financial Disclosures

AEMN: No disclosures.

DMK: No disclosures.

JKW: supported by NIH KL2TR001429.

MSO: serves as Medical Advisor the Parkinson’s Foundation, and has received research grants from NIH, Parkinson’s Foundation, the Michael J. Fox Foundation, the Parkinson Alliance, the Smallwood Foundation, the Tourette Association of America, and the UF Foundation. Dr. Okun’s research is supported by: R01 NS131342 NIH R01 NR014852, R01NS096008, UH3NS119844, U01NS119562. Dr. Okun is a multi-PI of the NIH R25NS108939 Training Grant. Dr. Okun has received royalties for publications with Hachette Book Group, Demos, Manson, Amazon, Smashwords, Books4Patients, Perseus, Robert Rose, Oxford and Cambridge (movement disorders books). Dr. Okun is an associate editor for New England Journal of Medicine Journal Watch Neurology and JAMA Neurology. Dr. Okun has participated in CME and educational activities (past 12–24 months) on movement disorders sponsored by WebMD/Medscape, RMEI Medical Education, American Academy of Neurology, Movement Disorders Society, Mediflix and by Vanderbilt University. Dr. Okun receive grants from industry. Dr. Okun has participated as a site PI and/or co-I for several NIH, foundation, and industry sponsored trials over the years but has not received honoraria. Research projects at the University of Florida receive device and drug donations.

JDH: has received consulting fees from AskBio.
